# A Novel Strategy for Screening Active Components in *Cistanche tubulosa* Based on Spectrum-Effect Relationship Analysis and Network Pharmacology

**DOI:** 10.1155/2023/9030015

**Published:** 2023-01-31

**Authors:** Xiao-Tong Liu, Dong-Mei Sun, Wen-Xin Yu, Wei-Xiong Lin, Liao-Yuan Liu, Yu Zeng

**Affiliations:** ^1^School of Chinese Materia Medica, Guangdong Pharmaceutical University, Guangzhou 510006, China; ^2^Guangdong Provincial Key Laboratory of Traditional Chinese Medicine Formula Granule, Guangdong E-Fong Pharmaceutical Co. Ltd., Foshan 528244, China

## Abstract

*Cistanche tubulosa* (Schenk) R. Wight is a valuable herbal medicine in China. The study aimed to explore the potential mechanisms of *C. tubulosa* on antioxidant activity using spectrum-effect relationship and network pharmacology and the possibilities of utilizing herbal dregs. In this work, different extracts of *C. tubulosa*, including herbal materials, water extracts, and herbal residues, were evaluated using high-performance liquid chromatography (HPLC) technology. In addition, the antioxidant activities were estimated *in vitro*, including 2, 2-diphenyl-1-picrylhydrazyl; superoxide anion; and hydroxyl radical scavenging assays. The spectrum-effect relationships between the HPLC fingerprints and the biological capabilities were analyzed via partial least squares regression, bivariate correlation analysis, and redundancy analysis. Furthermore, network pharmacology was used to predict potential mechanisms of *C. tubulosa* in the treatment of antioxidant-related diseases. According to the results, eleven common peaks were shared by different extracts. Geniposidic acid, echinacoside, verbascoside, tubuloside A, and isoacteoside were quantified and compared among different forms of *C. tubulosa*. The spectrum-effect relationship study indicated that peak *A*_6_ might be the most decisive component among the three forms. Based on network pharmacology, there were 159 target genes shared by active components and antioxidant-related diseases. Targets related to antioxidant activity and relevant pathways were discussed. Our results provide a theoretical basis for recycling the herbal residues and the potential mechanisms of *C. tubulosa* in the treatment of antioxidant-related diseases.

## 1. Introduction

*Cistanche tubulosa* (Schenk) R. Wight, one of the most frequently used herbs in the *Cistanche* family, is known as the “Ginseng of the Desert” for its various health benefits [1]. Modern pharmacological investigations have discovered that the *Cistanche* family offers various pharmaceutical effects, such as antioxidant, anticancer, hypoglycemic, antidepressant, cognitive improvement, and antimicrobial effects [2]. The primary chemical cluster in *Cistancheherb a* is phenylethanoid glycosides (PhGs) [1]. PhGs derived from *Cistanche* species can elevate the activities of superoxide dismutase (SOD) and glutathione peroxidase (GSH-Px) [3]. Echinacoside, one member of the PhG family, reverses effectively *in vivo* oxidative stress induced by high-glucose diets via downregulation of the nitrous oxide system (NOS) activity and phospho-eNOS expression [4]. The basic skeleton of the PhG consists of phenylethyl alcohol and glycosyl moieties. PhGs are believed to have a significant activity due to phenolic hydroxyl groups in their structure. The antioxidant activity of PhGs increases with the presence of phenolic hydroxyl groups [5].

Reactive oxygen species (ROS) have crucial roles in many physiological processes and essential protective mechanisms. Frequent exposure to high ROS concentrations may contribute to nonspecific damage to proteins, lipids, and nucleic acids. ROSs can be neutral molecules (e.g., hydrogen peroxide), ions (e.g., superoxide anion), or radicals (e.g., hydroxyl radicals) and exert their effects via regulation of cell signaling cascades [6]. Their rapid production and removal are influenced by a variety of mechanisms. They are lightweight and diffuse easily across short distances [7]. The superoxide anion (O_2_^•−^) is a precursor of most ROSs and an intermediate species in oxidative reactions. Hydroxyl radicals (OH•) are catalyzed by reduced transition metals, which may in turn be reoxidised by O_2_^•−^. An imbalance between excessive production of ROS and limited antioxidant defenses leads to various deleterious processes also called “oxidative stress” [8]. If such an imbalance can be corrected, the management of several defense mechanisms may be manipulated. Oxidative stress is involved in various pathological conditions, such as cancer, cardiovascular disease, neurological disorders, and diabetes [6]. In addition, the 2,2-diphenyl-1-picrylhydrazyl (DPPH•) radical is colored and remarkably stable, and thus is among the most common radicals considered in numerous studies [9]. A DPPH scavenging assay is an easy-to-implement, accurate method of measuring the total antioxidant capacities of botanical or herbal extracts [10]. Therefore, DPPH, superoxide anion, and hydroxyl radical scavenging assays have been used to evaluate the antioxidant capacity of different extracts of *C. tubulosa*.

The extract production steps for traditional Chinese medicine (TCM) are complex and involve cutting, processing, extraction, concentration, and drying [11]. It is inevitable that active components in herbal materials (HMs) will be lost during such sophisticated manufacturing stages. PhGs are characterized by at least one glycosyl moiety at their core, which determines their properties. These compounds are water soluble and easy to extract via traditional methods. It is also the case that water extraction technology is widely used in the production of TCM [12]. The resulting water extracts (WEs) are formulated into various dosage forms such as tablets, granules, capsules, and mixtures. Inevitably, the loss of biological ingredients occurs during such a large-scale production process. This is why quality assessment is an indispensable step and is required to ensure the quality of semifinished products from processes. Some researchers have already utilized these residual materials via structural modification. *Astragalus membranaceus* residue was purified to produce a polysaccharide that improved cognitive dysfunction by altering gut microbiota in diabetic mice [13]. A neutral polysaccharide extracted from *Codonopsis pilosula* residue exhibited a hypoglycemic effect [14]. Large amounts of herbal residues (HRs) are manufactured in China. The reutilization of HRs has become a new and novel research field as TCM processes have undergone modernization [15]. The potential for differences between the antioxidant properties of various herbs and their WEs has become a focus of our attention. In addition to considering WEs of *C. tubulosa*, it is hoped to determine whether HRs can prove useful as candidate TCMs and to explore opportunities for transforming discarded *C. tubulosa* waste material into feasible products.

Spectrum-effect relationships are utilized to determine effective components in complex mixtures and reflect the internal quality of herbal medicine. It is indispensable in the process of modernization and internationalization of herbal medicine. Since the spectrum-effect relationship research of herbal medicines is based on the chromatographic fingerprint, a suitable analytical method is required to generate a fingerprint that reflects the chemical ingredients of herbal medicines. High-performance liquid chromatography (HPLC) is an important analytical method that has many advantages, such as high separation, good stability, high efficiency, and high quantitative precision. PLSR (partial least squares regression) simplifies the data structure and correlation analysis between two sets of variables by using regression modeling [16]. In bivariate correlation analysis (BCA), test scores are correlated with conceptually related constructs in order to establish valid evidence [17]. RDA (redundancy analysis) has been used to identify the primary microbial communities related to special biological capacity, but we applied it to the spectrum-effect relationship [18]. To evaluate the correlation coefficients, PLSR and BCA were used. The results were then verified using RDA to determine which models were more appropriate for studying the spectrum-effect relationship with *C. tubulosa*.

In the methodologies of “multicomponent therapeutics, biological network” in network pharmacology, we try to search for common targets between active molecules and diseases, which may play an indispensable role in providing a reference for the prevention of diseases. *Cistanche herba* exhibit potential multicomponent and multitarget properties in the previous reports [19, 20]. Biomarkers of oxidative damage associated with human diseases are summarized [21]. Few studies clarify the antioxidative mechanism of *C. tubulosa*. Hence, the network pharmacology technology was adopted to investigate bioactive molecules of *C. tubulosa* and mechanisms of *C. tubulosa* against oxidation.

In this study, the chromatographic fingerprints and antioxidant activities of HMs, WEs, and HRs from 11 batches of *C. tubulosa* were evaluated simultaneously via HPLC and antioxidant assays. A spectrum-effect relationship between the HPLC fingerprints and the antioxidant effects of *C. tubulosa* was revealed clearly via a series of correlation analyses. Existing studies mainly report on the antioxidative properties of *C. tubulosa* herbs [22–24], but few of them highlight the antioxidant capabilities of the HEs. In addition, the role of HRs might be understated, and this study provides a new opportunity to take advantage of new research in this field. The purpose of this research was to identify the major active ingredients within and the antioxidant activities of HMs, WEs, and HRs of *C. tubulosa*. Then, chemometrics was applied to identify spectrum-effect relationships for the HMs, WEs, and HRs from 11 batches of *C. tubulosa*. This is the first time that differences among HMs, WEs, and HRs of *C. tubulosa* have been studied in this manner. In addition, the network pharmacology analysis was performed to elucidate the underlying mechanisms of *C. tubulosa* in the treatment of antioxidant-related diseases.

## 2. Materials and Methods

### 2.1. Materials and Reagents

Eleven batches of *Cistanche tubulosa* (S1–11) were supplied by the Guangdong E-Fong Pharmaceutical Co. Ltd. (Foshan, China). A hydroxyl radical scavenging capacity kit (number: ml076360, specification: 48T), superoxide anion radical scavenging capacity kit (number: G0129F, specification: 48T), and 1,1-Diphenyl-2-picrylhydrazyl radical scavenging capacity kit (number: JN6547, specification: 48T) were purchased from Shanghai Enzyme-linked Biotechnology Co., Ltd. (Shanghai, China); Suzhou Grace Biotechnology Co., Ltd. (Suzhou, China); and Shanghai Jining Industrial Co., Ltd. (Shanghai, China), respectively.

HPLC-grade methanol and acetonitrile were from Merck (Darmstadt, Germany), and HPLC-grade phosphoric acid was purchased from Tianjin Kemiou Chemical Reagent Co., Ltd. (Tianjin, China). Geniposidic acid (111828–201805, purity: 98.1%), tubuloside A (19051602, purity: 99.49%), and isoacteoside (19092702, purity: 98.58%) were purchased from Chengdu GLP Biotechnology Co., Ltd. (Chengdu, China). Verbascoside (111530–201914, purity: 95.2%) and echinacoside (111670–201907, purity: 91.8%) were purchased from the National Institutes for Food and Drug Control (Beijing, China). The water in the experimental studies was purified using a Merck Millipore purification system (Merck, Darmstadt, Germany). All other chemicals were of analytical grade.

### 2.2. Collection of WEs and HRs

**Figure 1:**
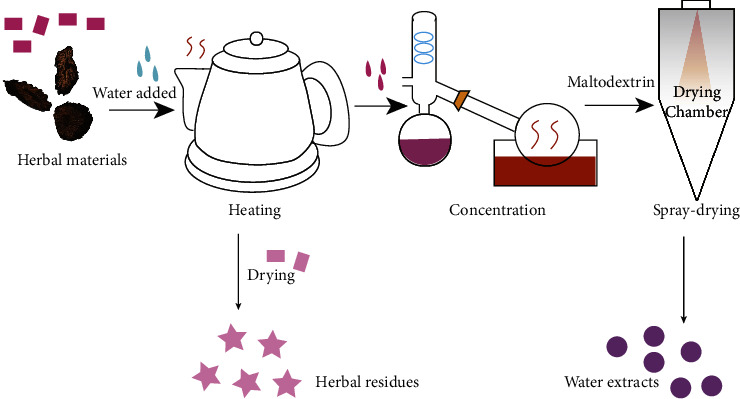
The procedure of processing raw materials of *C. tubulosa* into different forms.

WEs were prepared as described in the previous article with modifications [25–27]. Herbs (100 g) were extracted with water at a ratio of 1 : 10 (w/v) in a ceramic container and heated for 2 hours. After filtration, the extract and wet material were separated. The extraction process was repeated using water at a ratio of 1 : 8 (w/v) and the resulting material was heated for 1 hour. The two separate extracts were combined. The mixed extracts were concentrated using a rotary evaporator to yield a creamy solution with a density of 1.05 g/cm³. An appropriate amount of maltodextrin was added. Spray dryers have fast drying speeds and good product performance. Spraying atomizes the liquid material into dispersed particles, thereby increasing its surface area. Contacting hot air facilitates the drying process very quickly. A Buchi B-290 spray dryer (Buchi, Switzerland) was used to spray dry the mixture. The inlet and outlet temperatures were set to 175–205°C and 85–95°C, respectively. The WEs were collected. During processing of the WEs, the wet residues were collected and dried at 50°C to give the HRs.  Figure 1 shows the procedure for preparing WEs and HRs.

### 2.3. HPLC Condition

Chromatography was performed using a Vanquish™ Horizon UHPLC (Thermo Fisher Scientific, Massachusetts, USA) with an ultraviolet detector and an ACQUITY UPLC BEH C_18_ column (2.1 mm × 100 mm, 1.7 *μ*m) at 30°C. The mobile phase consisted of 0.1% phosphoric acid solution (A) in acetonitrile (B) in gradient elution mode as follows: 0–8 min 5%–13% B, 8-9 min 13%–15% B, 9–19 min 15% B, 19–20 min 15%-5% B, and 20–26 min 5% B. The flow rate was 0.3 mL/min, the sample injection volume was 1 *µ*L, and the detector wavelength was set to 238 nm.

### 2.4. Preparation of Samples and Standards for HPLC Analysis

#### 2.4.1. Preparation of Sample Solutions

Sample solutions were prepared according to Zhen et al. with modifications [28]. *Cistanche tubulosa* herbs and the dregs were ground into powder (65 mesh). The pulverized samples (1.0 g) were soaked for 30 min and extracted with 50 mL of 50% aqueous methanol in an ultrasonic bath for 40 min (250 W, 40 kHz). The solutions were then filtered through a 0.22-*μ*L microporous membrane. Then, the samples of WEs (0.2 g) were extracted with 50 mL of 50% aqueous methanol in an ultrasonic bath for 30 min (250 W, 40 kHz). The solutions were then filtered through a 0.22-*μ*L microporous membrane.

#### 2.4.2. Preparation of Standard Solutions

Appropriate amounts of five reference compounds were dissolved in 50% aqueous methanol and then filtered through a 0.22-*μ*L microporous membrane to yield a mixed standard solution. Upon adding 50% aqueous methanol to a 10-mL volumetric flask, the mixed standard solution contained geniposidic acid, echinacoside, verbascoside, tubuloside A, and isoacteoside. Pure reference compound solutions were injected into the HPLC system for qualitative analysis, and their retention times were recorded. Comparing retention times allowed reference compounds to be identified.

### 2.5. Methodology Validation

#### 2.5.1. Precision, Reproducibility, and Stability

The powder of HM1 was prepared as described in Section 2.4. Method precision was evaluated using six successive injections of one sample solution, while reproducibility was estimated by performing six replicates of a sample. Stability tests were performed by replicating injections of one sample solution that had been kept at 15°C for 0, 2, 4, 8, 12, and 24 h.

#### 2.5.2. Linearity

The mixed reference solutions with different gradient concentrations were injected and analyzed. The concentration ranges for geniposidic acid, echinacoside, verbascoside, tubuloside A, and isoacteoside were 0.0011–0.3414 mg/mL, 0.0081–2.421 mg/mL, 0.001–0.3132 mg/mL, 0.0005–0.1518 mg/mL, and 0.0004–0.1116 mg/mL, respectively. Analytical curves for each standard were obtained by considering the correlation between the peak area (*y*) and concentration (*x*, mg/mL) using a linear least squares model.

#### 2.5.3. Sample Recovery

Sample recovery was investigated by adding an accurate amount of standard solution to 0.5 g of HM1 sample powder. Nine samples were prepared in parallel according to Section 2.4. The mean sample recovery of each component was determined.

#### 2.5.4. Sample Determination

As described in Section 2.4, HMs, Wes, and HRs were prepared in parallel. These sample solutions were injected following the chromatographic conditions described in Section 2.3. The peak area was recorded and the contents were calculated. The data were analyzed using GraphPad Prism 8 (GraphPad Software, California, USA). *p* values were calculated using the one-way analysis of variance followed by the Tukey method. *p* < 0.05 was considered statistically significant.

### 2.6. Fingerprint Establishment and Evaluation

HPLC chromatographic data were output from Chromeleon 7.2.8 Software (Thermo Fisher Scientific, Massachusetts, USA) in CDF and TXT format. The HM-WE, WE-HR, and HM-HR similarity values were calculated using a similarity evaluation system designed for chromatographic fingerprints within TCM Software (Version 2004A). HPLC fingerprints were drawn using Origin 2021 Software (OriginLab, Massachusetts, USA).

### 2.7. Antioxidant Activity Evaluation

The measurement procedures for the antioxidant level were conducted according to the instructions provided in the various kits. The absorbance values were measured using a Shimadzu UV-2600i (Shimadzu, Japan). Each sample was run in triplicate, and the average data were recorded. Various sample concentrations and their corresponding absorbance (*A*) values were recorded. The 50% inhibiting concentration (IC_50_) was calculated using GraphPad Prism 8 (GraphPad Software, California, USA).

#### 2.7.1. DPPH Assay

First, 0.1 g of HM, WE, and HR powders were extracted using 1 mL of an 80% aqueous methanol solution. The resulting material was sonicated for 30 min and centrifuged at 12,000 × *g* for 5 min. A 400 *μ*L sample was mixed with the working fluids (Table S1), and a 1-mL cuvette was prepared. After the reaction in the dark at 25°C for 30 min, the absorbance at 517 nm was recorded and converted to radical scavenging activity (*S*_DPPH_) as follows:

(1) $$\begin{array}{c} S_{DPPH}\left(\%\right)=\left(1-\frac{A_{sample}-A_{control}}{A_{blank}}\right)\times 100\%\text{.} \end{array}$$

Dehydrated ethanol was used to adjust to zero.

#### 2.7.2. O_2_^•−^ Assay

First, 0.1 g of HM, WE, and HR powders were extracted using 1 mL of an 80% aqueous ethanol solution. The resulting material was sonicated for 30 min and centrifuged at 12,000 × *g* for 5 min. A 100 *μ*L sample was added into the working fluids (Table S2). After the reaction was allowed to proceed at 37°C for 10 min, the absorbance at 570 nm was recorded and converted to radical scavenging activity (*S*_*O*_2_^•−^_) as follows:

(2) $$\begin{array}{c} S_{O_{2}^{•-}}\left(\%\right)=\left(1-\frac{A_{sample}-A_{control}}{A_{blank}}\right)\times 100\%\text{.} \end{array}$$

Purified water was used to adjust to zero.

#### 2.7.3. OH• Assay

First, 0.1 g of HM, WE, and HR powders were extracted using 1 mL of diluent from the manufacturer's kit. The resulting material was sonicated for 30 min and centrifuged at 12,000 × *g* for 5 min. The working fluids were mixed quickly to avoid over color rendering. Then, a 250-*μ*L sample solution was transferred to the working fluids (Table S3). After incubation at 37°C for 20 min, the absorbance at 536 nm was recorded, and the radical scavenging activity (S_OH•_) was determined as follows:

(3) $$\begin{array}{c} S_{OH•}\left(\%\right)=\frac{A_{sample}-A_{control}}{A_{blank}-A_{control}}\times 100\%\text{.} \end{array}$$

Purified water was used to adjust to zero.

### 2.8. Data Analysis

#### 2.8.1. PLSR Analysis

The main chromatographic peak areas served as the independent variables (*X*) and the levels of antioxidant activity for the various assays were the dependent variables (*Y*). PLSR modeling was performed using Unscrambler *X* 10.4 Software (CAMO Software, Bangalore, India). The weighted regression coefficients revealed correlations between the peak areas and antioxidant activity levels, and the raw regression coefficients defined the model equation.

#### 2.8.2. BCA Analysis

Peak areas were the independent variables (*X*), and the antioxidant levels for the various assays were treated as the dependent variables (*Y*). Then, the BCA between *X* and *Y* was analyzed using a Pearson model. This procedure was performed using SPSS statistical software (SPSS for Windows 26.0, SPSS Inc., San Francisco, USA).

#### 2.8.3. Heatmap Chart, Venn Chart, and RDA

Heatmap charts were drawn using GraphPad Prism 8 (GraphPad Software, California, USA). Based on the PLSR and BCA models, the relationship values between the peak area and the antioxidant capabilities of 11 peaks were ranked from large to small, and the top five grades were recorded. These peaks were used to draw Venn diagrams online (https://bioinformatics.psb.ugent.be/webtools/Venn/). The data for the various peak areas and antioxidant levels for the three forms of *C. tubulosa* were visualized via RDA. The RDA tests were performed using CANOCO Software (Biometris—Plant Research International, Wageningen, The Netherlands).

### 2.9. Network Pharmacology Analysis

#### 2.9.1. Screening for Active Ingredients of *C. tubulosa*

All the chemical constituents of *C. tubulosa* were obtained using traditional Chinese medicine systems pharmacology (TCMSP, https://www.tcmsp-e.com/). The screening thresholds of each chemical component were set as oral bioavailability (OB) ≥ 30% and drug-likeness (DL) ≥ 0.18, respectively. The InChIKey of bioactive ingredients was collected through the PubChem database (https://pubchem.ncbi.nlm.nih.gov/). The protein targets of the active compounds were screened out through the SwissTargetPrediction database (https://www.swisstargetprediction.ch/). The target names were converted into gene names using the UniPort protein database (https://www.uniprot.org/).

#### 2.9.2. Construction of a Component-Target Network

The keyword “antioxidant” was used to search for disease-related targets on the GeneCards database (https://www.genecards.org/) and OMIM database (https://omim.org/). The intersections of genes between active components and disease-related targets were visualized using a Venn diagram online (https://bioinformatics.psb.ugent.be/webtools/Venn/). The bioactive ingredient targets of *C. tubulosa* were mapped to the target genes using Cytoscape 3.9.1 software (https://cytoscape.org/) for constructing the component-target (C-T) network.

#### 2.9.3. Gene Ontology and Kyoto Encyclopedia of Genes and Genomes Enrichment Analyses

Gene ontology (GO) enrichment in biological processes (BP), cellular component (CC), and molecular function (MF), and kyoto encyclopedia of genes and genomes (KEGG) pathway enrichment were analyzed online using the Metascape database (https://www.metascape.org/) with the “Homo sapiens” setting. The visualization bubble chart and GO histogram were formed online (https://www.bioinformatics.com.cn/).

#### 2.9.4. Establishment of Protein-Protein Interaction and Component-Target-Pathway Networks

The overlapping antioxidation-related and predicted targets from active components were used to construct a protein-protein interaction (PPI) using the STRING database (https://stringdb.org/). The conditions were set as described by Xin et al. [29]. The PPI network was visualized using the Cytoscape software. Degree centrality (DC), betweenness centrality (BC), and closeness centrality (CC) were calculated through the “network analysis” function. DC, BC, and CC were set as >100, >0.03, and >0.3, respectively. According to KEGG pathways and target genes, Cytoscape software was used to construct a component-target-pathway (C-T-P) network.

## 3. Results and Discussion

### 3.1. HPLC Fingerprints

#### 3.1.1. Method Validation

The validation for the HPLC method showed that the relative standard deviation (RSD) for method precision, reproducibility, and stability was less than 2.85% for the relative peak area (*n* = 11) and 0.77% for the relative retention time (*n* = 11). The precision of the same sample solution appeared within the range of 0.05–0.77% for relative time and 0.28–2.70% for the relative area of the common peaks. The reproducibility of the experiment was within the range of 0.03–0.20% for the relative time and 0.23–2.59% for the relative area of the common peaks. The sample stability was 0.09–0.24% for relative retention time and 0.75–2.85% for the relative area of the common peaks. These results indicated that the established fingerprint was satisfied. The linear relationships for geniposidic acid, echinacoside, acteoside, tubuloside A, and isoacteoside are shown in Table S4. The value of *R* square was 1.0000, indicating good linearity. The results of sample recovery showed that the average recoveries of geniposidic acid, echinacoside, acteoside, tubuloside A, and isoacteoside were 100.37%, 103.59%, 98.46%, 100.81%, and 101.19%, and the RSD for sample recoveries was less than 2.68%.

#### 3.1.2. Peak Area (PA) and Relative Retention Time (RRT)

**Figure 2:**
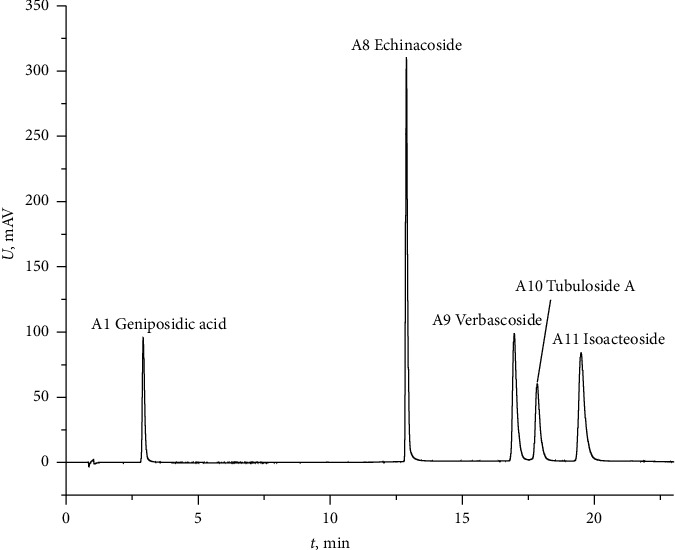
HPLC fingerprints of standard samples.

**Figure 3:**
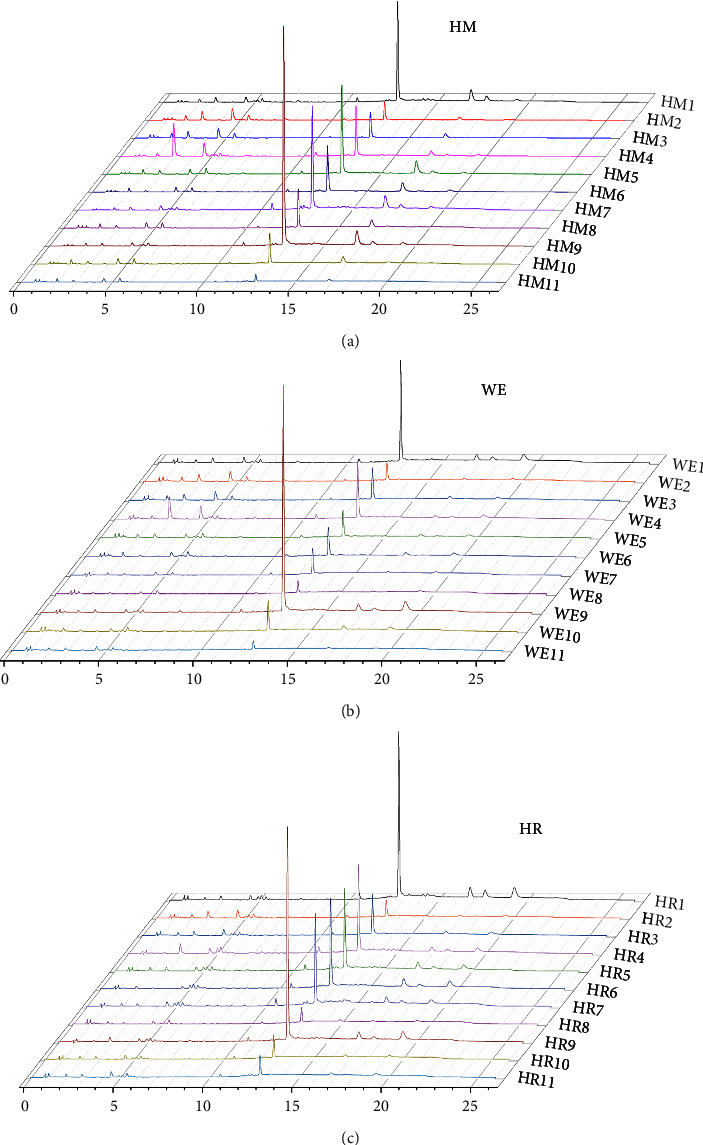
HPLC fingerprints of 11 batches of *C. tubulosa* samples.

The reference fingerprints and fingerprints of HMs, WEs, and HRs from 11 batches of *C. tubulosa* are presented in Figure 2. Eleven peaks, which exhibited good separation and resolution, were identified as common peaks among HMs, WEs, and HRs. The five standard compounds were identified as geniposidic acid (*A*_2_), echinacoside (*A*_8_), acteoside (*A*_9_), tubuloside A (*A*_10_), and isoacteoside (*A*_11_). The standard compound, echinacoside, which was present in all chromatograms (average retention time 12.86 min) with a suitable peak area and good stability, was selected as the reference peak and used to calculate the relative retention times (RRTs) of the other ten common peaks. The RRTs of these different forms are in the 0.16–1.51 range. The PA and coefficient of variance (CV%) of these common peaks are listed in Tables S5–S7. From the data, the CV% values for PA in various forms are 25.78%–142.02%, 23.36%–150.38%, and 28.91%–112.78% for HMs, WEs, and HRs, respectively. These results reveal significant differences in the concentration of each *Cistanche tubulosa* compound among the different forms. The fingerprints of HMs, WEs, and HRs are shown in Figure 3.

#### 3.1.3. Contents of HMs, WEs, and HRs

**Figure 4:**
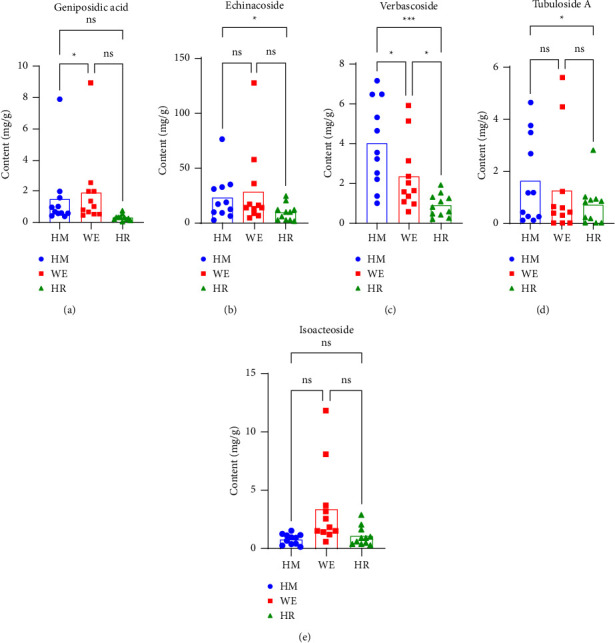
Content determination of five components from different forms (*n* = 11). ^*∗*^*p* < 0.05, ^*∗∗∗*^*p* < 0.001, ns: not significant.

Five standard constituents of *C. tubulosa* were measured. The contents of the main components are shown in Table 1. The comparison between HMs, WEs, and HRs is shown in Table 2 and Figure 4. PhGs in *C. tubulosa* are biologically active but thermosensitive. Heat-sensitive components dissolving in water can be efficiently extracted using a reasonable method. Generally, *Cistanche herba* is extracted with water and then evaporated into a concentrated solution for the following chemical analysis [26, 27, 30]. After extraction and concentration, spray-drying technology was used and the procedure was modified from the previous article. Water was quickly removed from the liquid steam, and then dry extracts of raw materials from plants were obtained. In this step, the addition of maltodextrin is considered as a common carrier to enhance the dispersion and extend the storage time. Through a series of manufacturing processes, herbal plants were then pressed into formula granules with additives. This step of adding excipients was not included in the experiment. Generally speaking, our production process includes extraction, concentration, and spray drying, as described in Section 2.2, in parallel with a formula granule production process. In order to produce these semifinished products, the above three steps must be followed. The procedure of forming WEs involves concentration and spray-drying, which easily cause the loss of thermosensitive components, but HRs are obtained after extraction and drying of HMs. We wonder whether it is possible that active components remain in HRs. According to our results, the content of verbascoside reduced significantly from HMs to WEs and HRs (*p* < 0.05 and *p* < 0.01, respectively). The thermal stability of verbascoside is investigated by monitoring the changes in the peak area through HPLC during the heating process. After heating for 4 h, 41.6% of verbascoside is left. It indicates that verbascoside is thermosensitive [31]. Isoacteoside, tubuloside A, and echinacoside in WEs remained stable after complex processing procedures. During the long-term drying process, the accumulation of PhGs showed a significant decrease, which might be attributed to the thermal degradation of these thermosensitive components [32]. In terms of the other target components, HRs and WEs did not differ significantly except for verbascoside. Our understanding of this difference will enable us to develop better quality standards for herbal dregs in the future and advance them into products.

#### 3.1.4. Fingerprint Similarity Analysis

The similarities among the three *C. tubulosa* groups were evaluated. The herbal material-water extract, herbal material-herbal residue, and water extract-herbal residue similarity values were in the ranges 0.943–0.994, 0.847–0.995, and 0.938–1.000, respectively (Table 3).

### 3.2. Antioxidant Activity Test Results

**Figure 5:**
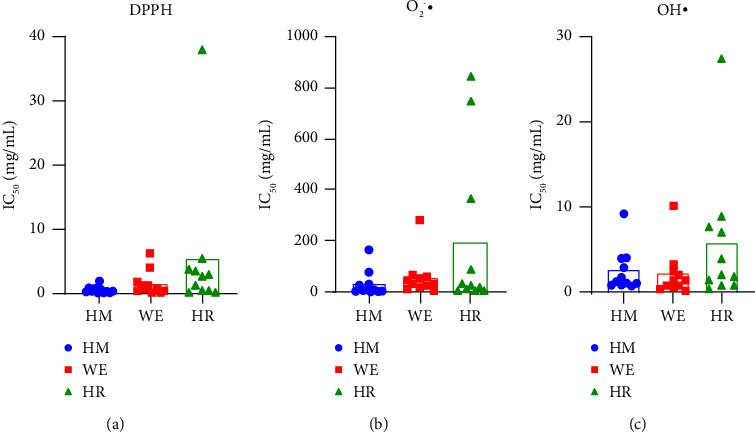
Column chart of IC50 of different forms for 11 batches of *C. tubulosa* samples.

The antioxidant activities of the various forms of *C. tubulosa* were determined using the DPPH, O_2_^•−^, and OH• scavenging capacity assays, and the relevant results are presented in Figure 5. In Table S8, the ranges for the DPPH, *O*_2_^•−^ and OH• scavenging capacity assay results were 0.04–37.80, 0.98–843.90, and 0.32–27.65 mg/mL for the three different forms among the 11 batches of *C. tubulosa*. In three antioxidant activity tests, HMs and WEs exhibited close inhibition activity, whereas HRs showed the weakest inhibition.

The spray-dried WEs were found to exhibit significant activities even at low concentrations. A previous report indicated that a spray-dried*Vernonia amygdalina* WE achieved 50% scavenging inhibition at 0.17 mg/mL [33]. The application of long extraction times and high temperatures is a double-edged sword. On the one hand, increasing the extraction time and spray drying inlet temperature improves the yield and efficiency. Moreover, the extracts achieve strong antioxidant activity and higher concentrations of biological components than those plants [34]. On the other hand, excessively hot inlet air degrades the bioactive compounds. Such elevated air inlet temperatures led to losses of antioxidant *Bidens pilosa* extract activity and were attributed to decreases in phenolic compounds [35]. Present results are consistent with the aforementioned report. For instance, the WE in S6 exhibited weaker radical inhibitory abilities than both HM and HR. Furthermore, HR in S5 exhibited stronger DPPH and superoxide anion scavenging abilities than HM and WE. The structure of PhGs consists of glycosidic bonds and acetyl groups that are hydrolyzed easily under enzymatic action or decomposed at high temperatures. These reactions may account for decreases in some main components during large-scale production. However, the hydrolysis or isomerization of certain components might accelerate the synthesis of other components. Such transformations are common when processing *Cistanches* herbs [36–38]. PhGs being water-soluble implies that most biological components can be utilized via water extraction. The contention that the majority of the active components remain in WEs has persisted for decades, so it seems reasonable to assume that the wet residual materials can be discarded after extraction. However, it is incorrect to regard HRs of *C. tubulosa* as waste. Researchers point out that PhGs are unstable, and they are susceptible to enzymatic or hydrolytic degradation [39]. Hydrolysis or isomerization reactions that contribute to decreases in biological ingredients within phytomedicines during processing might at the same time present new opportunities for exploiting HRs. By converting traditional extraction methods, medicinal residues can be developed and utilized more effectively. Enzymatic hydrolysis was performed to convert the *Panax ginseng* residue into monosugars. Yields of polysaccharides and ginsenosides increased, such as sugar, succinic acid, ginseng polysaccharides, and ginsenosides [40]. *Sophora flavescens* residues are reextracted by ultrasonic waves with ethyl acetate [41]. The updated technologies for utilizing herbal residues are summarized by Huang et al. [42].

### 3.3. Spectrum-Effect Relationship

**Figure 6:**
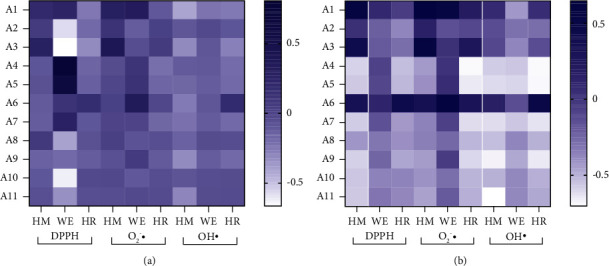
Heatmap diagrams: (a) PLSR model. (b) BCA model.

The spectrum-effect relationships between chromatographic peaks and antioxidant abilities were revealed using PLSR (regression equations obtained using the PLSR model can be seen in Supplementary Materials) and BCA models. The heatmap diagram was drawn to visualize the relationship (Figure 6). The relationship values and ranks are listed in Table 4.

**Figure 7:**
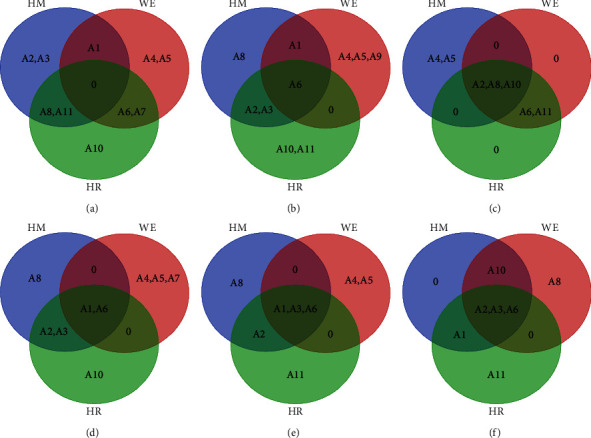
Venn diagrams of PLSR and BCA model: (a) DPPH assay. (b) O_2_^•−^ scavenging assay. (c) OH• scavenging assay were analyzed by the PLSR model. (d) DPPH assay. (e) O_2_^•−^ scavenging assay. (f) OH• scavenging assay were analyzed by the BCA model. The overlapping section was the common peaks shard by HM, WE, and HR.

**Figure 8:**
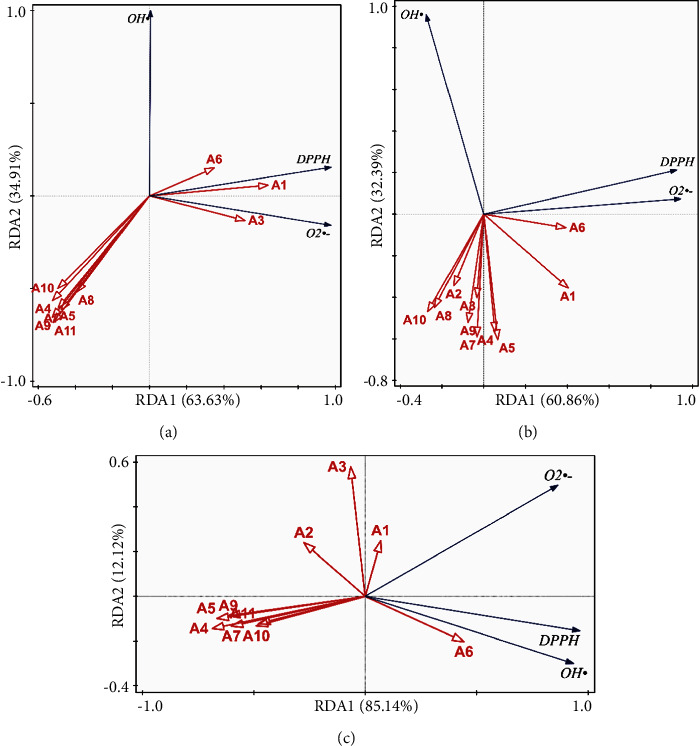
RDA between antioxidant ability and peaks: (a) HM of *C. tubulosa*. (b) WE of *C. tubulosa*. (c) HR of *C. tubulosa*. The intersection angle represents the relevance between the scavenging ability of the free radicals and the peak. The smaller the angle is, the more relevance there is with the peak of the antioxidant.

Based on the PLSR and BCA results, the top five peaks of different forms were screened using the DPPH, superoxide anion, and hydroxyl radical scavenging assays to identify the most important peaks. The results are illustrated in the Venn chart (Figure 7). *A*_2_, *A*_6_, *A*_8_, and *A*_10_ are the common peaks that are shared by HM, WE, and HR (Figures 7(a) and 7(c)) in the superoxide anion and hydroxyl radical scavenging assays, whereas HM, WE, and HR share no DPPH assay peaks. Meanwhile, the BCA models show that *A*_1_, *A*_2_, *A*_3_, and *A*_6_ are the common peaks shared by HM, WE, and HR (Figures 7(d)–7(f)). Notably, the overlaps in the Venn diagram indicate that the BCA model appears more suitable than the PLSR model, the former exhibiting more repetition. The BCA model coefficients and antioxidant ability IC_50_ values were analyzed via RDA. As the RDA shown in Figure 8, *A*_1_, *A*_3_, and *A*_6_ from HM and HR are related positively to the antioxidant indexes, except that *A*_3_ is related negatively to the hydroxyl radical scavenging capacity. *A*_1_ and *A*_6_ from WE have strong correlations with DPPH and the superoxide anion. The *A*_6_ peaks noted from the various forms exhibit the strongest connection to the DPPH, superoxide anion, and hydroxyl radicals. *A*_1_ and *A*_3_ also exhibit a similar connection.

### 3.4. Network Pharmacology-Based Analysis

#### 3.4.1. Construction of C-T Network

**Figure 9:**
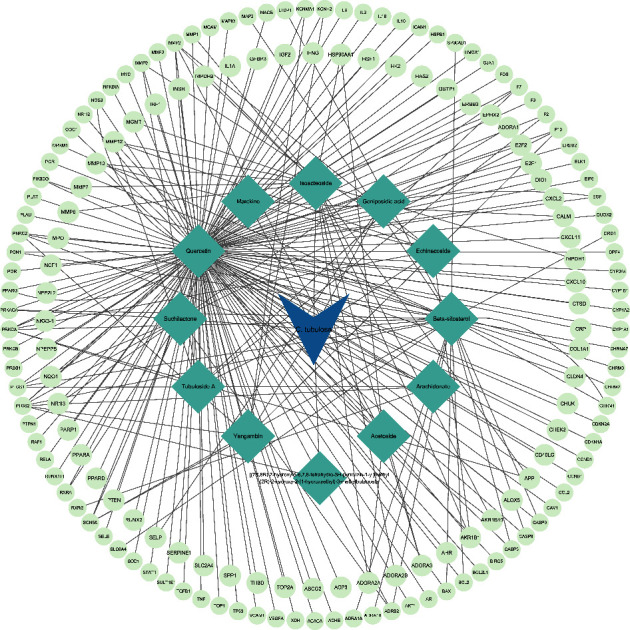
C-T network. The network showed the correlation between active components and the key gene targets.

A total of 4359 targets related to the antioxidant activity were obtained from the GeneCards database and the OMIM database. At the same time, active components were screened from the TCMSP database and the SwissTargetPrediction database. Then, 198 targets were collected and standardized through the UniPort database. There were 159 target genes shared by active components and antioxidant-related diseases (see Figure S1). The C-T network was constructed to illustrate the correlation between the compounds and the key gene targets (Figure 9).

#### 3.4.2. Construction of the PPI Network and Screening of Key Targets

**Figure 10:**
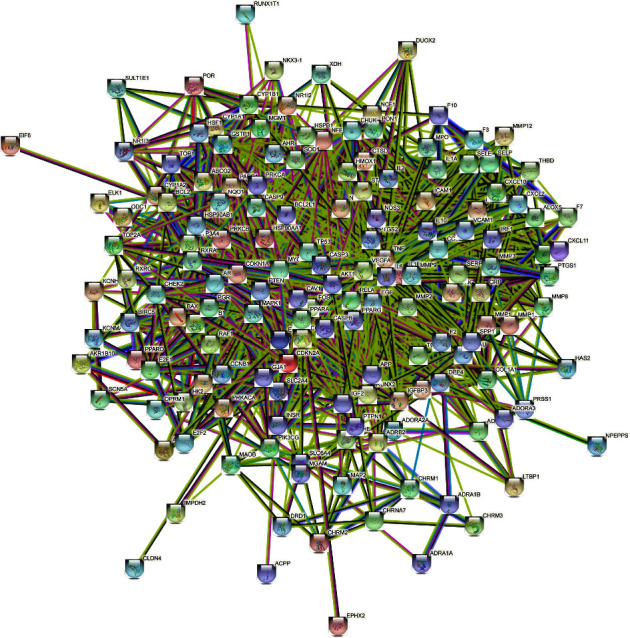
PPI network.

**Figure 11:**
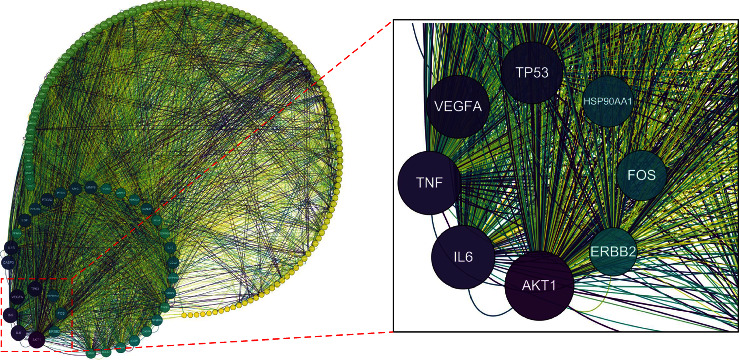
The key targets were screened out according to DC, CC, and BC.

PPI was visualized using the STRING database (Figure 10). The network included 159 nodes and 2528 edges. In the entire interaction network, the connecting components or the nodes with more target points may be the key component or target gene that plays an antioxidant role in *C. tubulosa*. The results were downloaded and introduced into Cytoscape for visualization. The higher the DC value, the darker the color, and the larger the combined score value, the thicker the edge. We found that RAC-alpha serine/threonine-protein kinase (AKT1), interlukin-6 (IL6), tumor necrosis factor (TNF), and vascular endothelial growth factor A (VEGFA) were centrally located (Figure 11), indicating that they were key targets when active components exerted an antioxidant effect. It is reported that echinacoside reduces mitochondrial dysfunction via regulation of mitogen-activated protein kinases (MAPK) and AKT and their phosphorylated forms [43]. Researchers speculated that the antidiabetic effect of glycosides of *C. tubulosa* might be due to the antioxidant activity of PhGs by downregulating proinflammatory cytokines, such as IL-6 and TNF-*α* [44]. In addition, echinacoside could impair ovarian cancer cell growth by downregulating the expression of VEGFA to inhibit angiogenesis [45], which is closely correlated to the ROS system for ROS induces the expression of VEGF signaling [46].

#### 3.4.3. Enrichment Analysis and C-T-P Network Establishment

**Figure 12:**
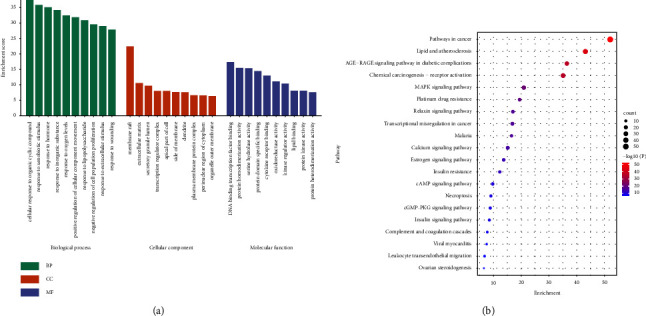
Enrichment analysis: (a) GO enrichment analysis. (b) KEGG enrichment analysis.

The potential antioxidant compounds acted on numerous biological functions, including BP, CC, and MF. In Figure 12(a), the top 10 pathways are shown. The predicted targets from the PPI network mainly responded to many biological processes, such as organic cyclic compounds, xenobiotic stimulus, inorganic substances, oxygen levels, and positive regulation of the cellular component movement. The cellular component analysis showed that the genes were mainly related to the membrane raft, extracellular matrix, secretory granule lumen, transcription regulator complex, and apical part of the cell. These targets are also involved in many molecular functions, including DNA-binding transcription factor binding, protein homodimerization activity, protein domain-specific binding, and cytokine receptor binding.

To investigate the biological functions of these major hubs, a pathway enrichment analysis was conducted. From KEGG enrichment results, a bubble diagram was drawn to show top 20 pathways. The larger the spot was, the more genes were included in the pathway. As shown in Figure 12(b), the key pathways of *C. tubulosa* were related to pathways in cancer, lipid and atherosclerosis, AGE-RAGE signaling pathway in diabetic complications, chemical carcinogenesis—receptor activation, and MAPK signaling pathway. Effects of *C. tubulosa* on apoptosis and cellular redox homeostasis were investigated. The data suggest that *C. tubulosa* can be a promising candidate for anti-colon-cancer therapy [47]. *C. deserticola* extract is found in aged people [48].

**Figure 13:**
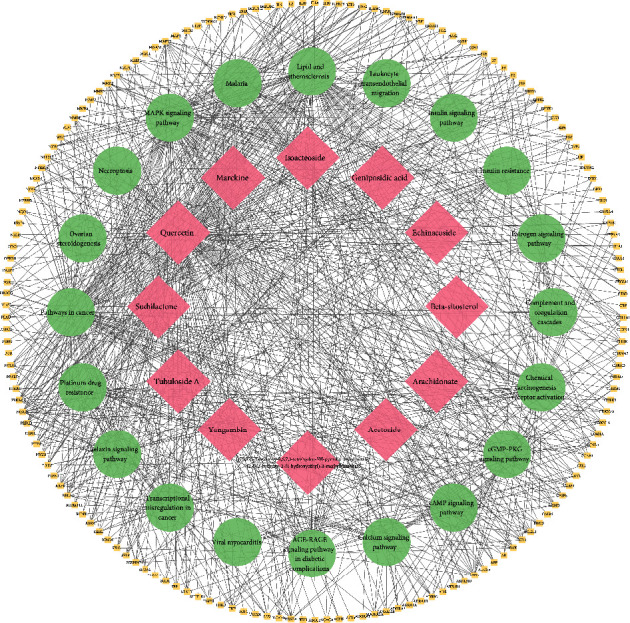
C-T-P network.

Figure 13 illustrates the correlation between the pathways and their related targets and the relationship between the overlapping target genes and biologically active components of *C. tubulosa*. A global view of the C-T-P network was generated, which consisted of 12 ingredients, 159 targets, and 20 pathways. Most of the targets were shared by the candidate active compounds. These candidate active ingredients with high interconnection degrees were responsible for the high interconnectedness of the C-T-P network, especially quercetin (degree = 131). The majority of the targets, such as AKT1, IL6, TNF, and VEGFA, were mapped to KEGG pathways associated with pathways in cancer.

## 4. Conclusions

In this study, we primarily probed complex situations when considering the spectrum-effect relationships among HM, WE, and HR of *C. tubulosa*. The HPLC fingerprints and antioxidant assays were used to identify the differences between Hs, WEs, and HRs of *C. tubulosa*. According to the HPLC fingerprints, 11 peaks were common among the 11 batches of Hs, WEs, and HRs. Geniposidic acid, echinacoside, verbascoside, tubuloside A, and isoacteoside were identified among these peaks. The contents of these five components were determined. In addition, the antioxidant effects of the *C. tubulosa* Hs, WEs, and HRs varied due to the alterations in the chemical compositions caused by complex manufacturing conditions. Based on diversified statistical models, the spectrum-effect relationship study indicated that peak *A*_6_ might be the most decisive component among the three forms of *C. tubulosa*. The study was based on network pharmacology to explore potential mechanisms of *C. tubulosa* on antioxidation through screening of compounds, prediction of key targets, construction of networks, and conduction of enrichment analysis. Our results provide a theoretical basis for recycling the herbal residues and the potential of *C. tubulosa* in the treatment of antioxidant-related diseases.

## Figures and Tables

**Table 1:** Contents of 11 batches of *C. tubulosa*.

	HM					WE					HR				
	Geniposidic acid (mg/g)	Echinacoside (mg/g)	Verbascoside (mg/g)	Tubuloside A (mg/g)	Isoacteoside (mg/g)	Geniposidic acid (mg/g)	Echinacoside (mg/g)	Verbascoside (mg/g)	Tubuloside A (mg/g)	Isoacteoside (mg/g)	Geniposidic acid (mg/g)	Echinacoside (mg/g)	Verbascoside (mg/g)	Tubuloside A (mg/g)	Isoacteoside (mg/g)
S1	1.02	30.54	5.30	4.64	1.10	1.98	57.58	5.13	5.58	8.09	0.34	20.09	1.92	2.81	2.92
S2	1.99	5.99	1.34	0.11	0.24	2.51	8.59	1.07	—	1.39	0.55	2.05	0.23	—	0.37
S3	1.58	8.80	2.19	0.25	0.41	2.01	15.85	2.02	0.60	1.85	0.34	5.16	0.58	0.17	0.60
S4	7.89	18.43	2.50	1.17	0.90	8.96	35.41	1.61	1.21	3.72	0.77	11.15	0.81	0.86	0.95
S5	0.97	32.31	6.46	2.67	0.95	1.38	13.01	1.58	0.43	1.52	0.30	9.74	1.22	0.87	1.00
S6	0.39	16.82	4.62	0.26	1.15	0.45	14.00	2.31	0.29	2.50	0.06	11.62	1.50	0.81	1.63
S7	0.69	34.69	6.45	3.75	1.24	0.70	13.83	1.33	0.63	1.53	0.19	9.99	1.08	1.03	0.92
S8	0.60	12.00	3.53	1.18	0.37	0.50	6.53	0.57	—	1.15	0.12	1.70	0.17	—	0.32
S9	0.59	75.95	7.14	3.48	1.52	1.03	127.43	5.89	4.46	11.80	0.34	24.70	1.30	0.93	2.08
S10	0.58	9.43	3.21	0.42	0.66	0.53	16.35	3.12	0.39	3.19	0.22	2.72	0.43	0.23	0.44
S11	0.42	2.18	0.98	0.11	0.13	0.79	4.55	0.92	—	0.62	0.25	2.28	0.38	—	0.41

**Table 2:** Comparison of contents of main components (*n* = 11).

Components	HM	WE	HR
Geniposidic acid (mg/g)	1.52 ± 2.17^c^	1.9 ± 2.45^a^	0.32 ± 0.2
Echinacoside (mg/g)	22.47 ± 20.9	28.47 ± 36.2	9.2 ± 7.64^a^
Verbascoside (mg/g)	3.97 ± 2.16^c^	2.32 ± 1.73^a^	0.87 ± 0.57^b^
Tubuloside A (mg/g)	1.64 ± 1.68	1.24 ± 1.92	0.7 ± 0.81^a^
Isoacteoside (mg/g)	0.79 ± 0.45	3.4 ± 3.46	1.06 ± 0.83

^a^indicates *p* < 0.05 versus HM, ^b^indicates *p* < 0.001 versus HM, and ^c^indicates *p* < 0.05 versus WE.

**Table 3:** Similarities of HM-WE, HM-HR, and WE-HR for 11 batches of *C. tubulosa*.

Samples	Batches										
	S1	S2	S3	S4	S5	S6	S7	S8	S9	S10	S11
HM-WE	0.985	0.989	0.981	0.970	0.963	0.974	0.973	0.943	0.994	0.967	0.959
HM-HR	0.984	0.974	0.933	0.847	0.989	0.972	0.991	0.934	0.995	0.966	0.898
WE-HR	0.998	0.999	0.979	0.938	0.973	0.968	0.988	0.997	1.000	0.983	0.979

**Table 4:** Correlation coefficients of PLSR and BCA models for the peak area and antioxidant assay.

Groups	Peaks	DPPH				O_2_^•−^				*OH*•			
		PLSR	Ranks	BCA	Ranks	PLSR	Ranks	BCA	Ranks	PLSR	Ranks	BCA	Ranks
H	A1	0.204	2	0.602	1	0.288	2	0.645	1	−0.413	11	0.070	2
	A2	0.058	4	0.036	4	0.122	3	0.154	4	−0.096	2	−0.133	4
	A3	0.282	1	0.418	2	0.387	1	0.588	2	−0.319	9	−0.111	3
	A4	−0.084	7	−0.586	11	−0.037	9	−0.435	9	−0.112	3	−0.570	7
	A5	−0.069	6	−0.564	8	−0.012	7	−0.399	7	−0.171	5	−0.609	8
	A6	−0.112	9	0.334	3	0.022	5	0.345	3	−0.257	7	0.162	1
	A7	−0.124	10	−0.574	9	0.007	6	−0.358	6	−0.217	6	−0.614	9
	A8	0.069	3	−0.418	5	0.029	4	−0.343	5	−0.148	4	−0.533	6
	A9	−0.148	11	−0.583	10	−0.064	11	−0.441	10	−0.321	10	−0.669	10
	A10	−0.092	8	−0.559	6	−0.022	8	−0.407	8	−0.079	1	−0.501	5
	A11	−0.059	5	−0.563	7	−0.047	10	−0.450	11	−0.300	8	−0.702	11

WE	A1	0.246	4	0.089	2	0.331	2	0.571	2	−0.231	11	−0.423	7
	A2	−0.578	9	−0.220	7	−0.124	11	−0.148	8	−0.029	1	−0.289	2
	A3	−0.666	11	−0.197	6	−0.044	8	0.023	4	−0.099	6	−0.365	3
	A4	0.807	1	−0.076	3	0.112	4	0.013	5	−0.108	7	−0.558	10
	A5	0.644	2	−0.102	4	0.127	3	0.053	3	−0.127	10	−0.593	11
	A6	0.114	5	0.115	1	0.343	1	0.605	1	−0.098	5	−0.137	1
	A7	0.274	3	−0.157	5	0.009	6	−0.072	7	−0.120	8	−0.557	9
	A8	−0.401	8	−0.315	10	−0.073	9	−0.258	10	−0.040	3	−0.366	4
	A9	−0.184	6	−0.244	8	0.065	5	−0.045	6	−0.125	9	−0.471	8
	A10	−0.626	10	−0.357	11	−0.103	10	−0.282	11	−0.044	4	−0.375	5
	A11	−0.342	7	−0.307	9	−0.030	7	−0.199	9	−0.038	2	−0.377	6

HR	A1	−0.249	10	−0.049	2	−0.102	7	0.199	3	−0.251	10	0.053	2
	A2	−0.186	9	−0.369	5	0.023	2	−0.107	4	−0.087	5	−0.276	4
	A3	−0.323	11	−0.275	3	0.060	1	0.256	2	−0.283	11	−0.134	3
	A4	−0.158	8	−0.533	11	−0.215	11	−0.688	11	−0.201	9	−0.689	11
	A5	−0.136	7	−0.518	10	−0.164	9	−0.647	10	−0.186	8	−0.683	10
	A6	0.170	1	0.430	1	−0.017	3	0.287	1	0.180	1	0.499	1
	A7	−0.090	5	−0.430	8	−0.172	10	−0.614	9	−0.183	6	−0.632	8
	A8	−0.068	4	−0.399	7	−0.048	6	−0.513	7	−0.043	4	−0.499	7
	A9	−0.128	6	−0.466	9	−0.117	8	−0.599	8	−0.184	7	−0.645	9
	A10	−0.028	2	−0.360	4	−0.046	5	−0.509	6	−0.038	3	−0.495	6
	A11	−0.052	3	−0.370	6	−0.035	4	−0.491	5	−0.031	2	−0.473	5

## Data Availability

All data generated or analyzed during this study are included in this paper.

## Abbreviations

HMs: Herbal materials
WEs: Water extracts
HRs: Herbal residues
PhGs: Phenylethanoid glycosides
SOD: Superoxide dismutase
GSH-Px: Glutathione peroxidase
NOS: Nitrous oxide system
ROS: Reactive oxygen species
DPPH: 2,2-diphenyl-1-picrylhydrazyl
O_2_^•−^: Superoxide anion
OH•: Hydroxyl radicals
TCM: Traditional Chinese medicine
HPLC: High-performance liquid chromatography
PA: Peak area
RRT: Relative retention time
RSD: Relative standard deviation
PLSR: Partial least squares regression
BCA: Bivariate correlation analysis
RDA: Redundancy analysis
IC_50_: Half inhibiting concentration
TCMSP: Traditional Chinese medicine systems pharmacology
GO: Gene ontology
KEGG: Kyoto encyclopedia of genes and genomes
BP: Biological process
CC: Cellular component
MF: Molecular function
DC: Degree centrality
CC: Closeness centrality
BC: Betweenness centrality
PPI: Protein-protein interaction
C-T: Component-targetnetwork
C-T-P: Component-target-pathway
AKT1: RAC-alpha serine/threonine-protein kinase
IL6: Interlukin 6
TNF: Tumor necrosis factor
VEGFA: Vascular endothelial growth factor A
AGE-RAGE: Accumulating evidence that advanced glycation end products-receptor for AGE.

## Supplementary Materials

Supplementary Materials Tables S1–S3 shows the experimental procedures for antioxidant assays. HPLC linearity results are shown in Table S4. In Tables S5–S7, peak areas for HMs, Wes, and HRs are displayed, see Table S8 for IC_50_ data. This file contains the PLSR equation. Figure S1 indicates key targets between *C. tubulosa* and diseases-related targets.
