# Supplementary material for: A Novel Strategy for Screening Active Components in Cistanche tubulosa Based on Spectrum-Effect Relationship Analysis and Network Pharmacology
Source: J Anal Methods Chem. 2023 Jan 31;2023:9030015. doi: 10.1155/2023/9030015 (PMC9904937; doi:10.1155/2023/9030015)
Supplement: Supplementary Materials — Tables S1–S3 shows the experimental procedures for antioxidant assays. HPLC linearity results are shown in Table S4. In Tables S5–S7, peak areas for HMs, Wes, and HRs are displayed, see Table S8 for IC50 data. This file contains the PLSR equation. Figure S1 indicates key targets between C. tubulosa and diseases-related targets. [file 9030015.f1.docx]

**Table of contents：**

1. Table S1 Instructions for DPPH scavenging assay kit provided by Shanghai JINING SHIYE Co., Ltd. (Shanghai, China)
2. Table S2 Instructions for *O*_2_^•-^ scavenging assay kit provided by Suzhou Grace Biotechnology Co., Ltd. (Suzhou, China)
3. Table S3 Instructions for *OH*• scavenging assay kit provided by Shanghai Mlbio Co., Ltd. (Shanghai, China)
4. Table S4 Regression equation and linear range
5. Table S5 The peak area of HM for 11 batches of *C. tubulosa*
6. Table S6 The peak area of WE for 11 batches of *C. tubulosa*
7. Table S7 The peak area of HR for 11 batches of *C. tubulosa*
8. Table S8 IC_50_ of antioxidant assays of different forms for 11 batches of *C. tubulosa* samples
9. The regression equations obtained using the PLSR model
10. Figure S1: Venn diagram. There were 159 key gene targets between *C. tubulosa*-related targets and antioxidant-related targets.

Table S1 Instructions for DPPH scavenging assay kit provided by Shanghai JINING SHIYE Co., Ltd (Shanghai, China).

| Solution type (μL) | Sample | Control | Blank |
| --- | --- | --- | --- |
| Sample solution | 400 | 400 |  |
| 80% aqueous methanol solution |  | 600 | 400 |
| Working fluid | 600 |  | 600 |
| After 30 min, the absorbance was measured. | | | |

Table S2 Instructions for *O*_2_^•-^ scavenging assay kit provided by Suzhou Grace Biotechnology Co., Ltd (Suzhou, China).

| Solution type (μL) | Sample | Control | Blank |
| --- | --- | --- | --- |
| Sample solution | 100 | 100 |  |
| Working fluid A | 440 | 480 | 540 |
| Working fluid B | 160 | 160 | 160 |
| Working fluid C | 40 |  | 40 |
| Working fluid D | 60 | 60 | 60 |
| After incubation at 37ºC for 10 min, the absorbance was measured. | | | |

Table S3 Instructions for *OH*• scavenging assay kit provided by Shanghai Mlbio Co., Ltd (Shanghai, China).

| Solution type (μL) | Sample | Control | Blank |
| --- | --- | --- | --- |
| Working fluid A | 150 | 150 | 150 |
| Working fluid B | 400 | 400 | 400 |
| Working fluid C | 100 | 100 | 100 |
| Quickly shaking to avoid over color rendering | | | |
| Sample | 250 |  |  |
| Working fluid D | 100 | 100 |  |
| H_2_O |  | 250 | 350 |
| After incubation at 37ºC for 1 h, the absorbance was measured. | | | |

Table S4 Regression equation and linear range

| Components | Regression equation | Linear relation R² | Linear range (mg/mL) |
| --- | --- | --- | --- |
| Geniposidic acid | y = 75.494x - 0.012 | 1.0000 | 0.0011-0.3424 |
| Echinacoside | y = 40.828x + 0.1579 | 1.0000 | 0.081-2.421 |
| Verbascoside | y = 54.641x + 0.0027 | 1.0000 | 0.001-0.3132 |
| Tubuloside A | y = 30.634x + 0.0042 | 1.0000 | 0.0005-0.3132 |
| Isoacteoside | y = 52.468x - 0.0053 | 1.0000 | 0.0004-0.1116 |

Table S5 The peak area of HM for 11 batches of *C. tubulosa*

|  | Peak area | | | | | | | | | | |
| --- | --- | --- | --- | --- | --- | --- | --- | --- | --- | --- | --- |
| Sample | A1 | A2-Geniposidic acid | A3 | A4 | A5 | A6 | A7 | A8-Echinacoside | A9-Verbascoside | A10-Tubuloside A | A11-Isoacteoside |
| H1 | 0.8624 | 1.6384 | 1.9393 | 0.4261 | 0.5547 | 1.1440 | 1.0669 | 26.8403 | 6.2019 | 3.0540 | 1.2428 |
| H2 | 1.3376 | 3.1477 | 4.3456 | 0.0909 | 0.1432 | 1.7127 | 0.4534 | 5.1620 | 1.5402 | 0.0730 | 0.2703 |
| H3 | 1.4278 | 2.4044 | 3.6235 | 0.0927 | 0.1671 | 1.5570 | 0.4005 | 7.3007 | 2.4242 | 0.1537 | 0.4353 |
| H4 | 0.8117 | 12.0965 | 4.9015 | 0.3665 | 0.5118 | 0.9223 | 1.1907 | 15.4002 | 2.7839 | 0.7336 | 0.9648 |
| H5 | 1.3105 | 1.5104 | 1.7616 | 0.2053 | 0.2814 | 1.8372 | 0.8541 | 27.5400 | 7.3390 | 1.7076 | 1.0427 |
| H6 | 1.1175 | 0.5881 | 1.7676 | 0.2386 | 0.3393 | 1.3555 | 0.8183 | 13.9456 | 5.0975 | 0.1595 | 1.2194 |
| H7 | 0.6290 | 1.0566 | 1.6927 | 0.3785 | 0.4837 | 0.8204 | 1.7398 | 29.1265 | 7.2189 | 2.3608 | 1.3344 |
| H8 | 1.1883 | 0.9203 | 2.0215 | 0.0996 | 0.1688 | 1.7263 | 0.4940 | 9.9995 | 3.9189 | 0.7397 | 0.4002 |
| H9 | 0.7086 | 0.9080 | 1.1952 | 0.3578 | 0.4744 | 0.9665 | 0.9561 | 63.7887 | 7.9846 | 2.1875 | 1.6355 |
| H10 | 1.1585 | 0.8743 | 1.9608 | 0.1071 | 0.1655 | 1.6901 | 0.3902 | 7.8082 | 3.5427 | 0.2634 | 0.7014 |
| H11 | 0.9433 | 0.6412 | 1.3168 | 0.0807 | 0.1106 | 1.2425 | 0.2230 | 1.8141 | 1.0820 | 0.0662 | 0.1437 |
| CV% | 25.777 | 142.024 | 52.486 | 61.730 | 54.921 | 26.812 | 57.360 | 92.960 | 54.741 | 103.886 | 57.671 |

Table S6 The peak area of WE for 11 batches of *C. tubulosa*

| Sample | Peak area | | | | | | | | | | |
| --- | --- | --- | --- | --- | --- | --- | --- | --- | --- | --- | --- |
|  | A1 | A2-Geniposidic acid | A3 | A4 | A5 | A6 | A7 | A8-Echinacoside | A9-Verbascoside | A10-Tubuloside A | A11-Isoacteoside |
| WE1 | 0.2873 | 0.5974 | 0.6989 | 0.1446 | 0.2089 | 0.3925 | 0.3379 | 9.4707 | 1.1231 | 0.6881 | 1.7076 |
| WE2 | 0.4262 | 0.9211 | 1.3603 | 0.0698 | 0.1103 | 0.5419 | 0.2199 | 1.7180 | 0.2841 | 0.0000 | 0.3565 |
| WE3 | 0.4598 | 0.7389 | 1.1813 | 0.0839 | 0.1382 | 0.4888 | 0.1992 | 3.1804 | 0.5407 | 0.0908 | 0.4757 |
| WE4 | 0.3025 | 2.8563 | 1.7162 | 0.1934 | 0.2742 | 0.3681 | 0.4627 | 6.1526 | 0.3732 | 0.1572 | 0.8287 |
| WE5 | 0.3574 | 0.5154 | 0.4920 | 0.0751 | 0.1227 | 0.4895 | 0.2170 | 2.6559 | 0.4284 | 0.0662 | 0.3989 |
| WE6 | 0.4245 | 0.1689 | 0.5736 | 0.1076 | 0.1591 | 0.5423 | 0.2020 | 2.8496 | 0.6251 | 0.0436 | 0.6541 |
| WE7 | 0.2222 | 0.2200 | 0.4015 | 0.0756 | 0.1013 | 0.3173 | 0.2122 | 2.3626 | 0.3021 | 0.0812 | 0.3347 |
| WE8 | 0.1722 | 0.1556 | 0.2533 | 0.0377 | 0.0504 | 0.3531 | 0.1012 | 1.1102 | 0.1302 | 0.0000 | 0.2507 |
| WE9 | 0.2126 | 0.3253 | 0.3699 | 0.1619 | 0.2211 | 0.2846 | 0.3032 | 21.8474 | 1.3441 | 0.5725 | 2.5962 |
| WE10 | 0.3167 | 0.1656 | 0.3444 | 0.0433 | 0.0657 | 0.4578 | 0.0914 | 2.7832 | 0.7085 | 0.0492 | 0.6965 |
| WE11 | 0.2064 | 0.2388 | 0.4127 | 0.0482 | 0.0660 | 0.2945 | 0.0926 | 0.7466 | 0.2008 | 0.0000 | 0.1304 |
| CV% | 32.274 | 124.806 | 68.547 | 54.355 | 51.931 | 23.365 | 50.894 | 122.657 | 69.502 | 150.375 | 96.769 |

Table S7 The peak area of HR for 11 batches of *C. tubulosa*

| Sample | Peak area | | | | | | | | | | |
| --- | --- | --- | --- | --- | --- | --- | --- | --- | --- | --- | --- |
|  | A1 | A2-Geniposidic acid | A3 | A4 | A5 | A6 | A7 | A8-Echinacoside | A9-Verbascoside | A10-Tubuloside A | A11-Isoacteoside |
| HR1 | 0.2440 | 0.5177 | 0.5848 | 0.3405 | 0.4782 | 0.3630 | 0.6054 | 16.4471 | 2.0886 | 1.7219 | 3.0635 |
| HR2 | 0.3488 | 0.8558 | 1.1402 | 0.1787 | 0.2514 | 0.4350 | 0.2580 | 1.7221 | 0.2632 | 0.0000 | 0.4023 |
| HR3 | 0.3035 | 0.5398 | 0.8912 | 0.1476 | 0.2179 | 0.3060 | 0.2456 | 4.5120 | 0.6719 | 0.1137 | 0.6713 |
| HR4 | 0.1205 | 1.2469 | 0.7109 | 0.4721 | 0.6434 | 0.1573 | 0.6745 | 9.8469 | 0.9491 | 0.5689 | 1.0787 |
| HR5 | 0.3572 | 0.4797 | 0.5066 | 0.3540 | 0.4211 | 0.4555 | 0.6061 | 8.5574 | 1.4235 | 0.5717 | 1.1248 |
| HR6 | 0.2537 | 0.0871 | 0.3244 | 0.3424 | 0.4636 | 0.3493 | 0.4665 | 9.9301 | 1.7047 | 0.5163 | 1.7913 |
| HR7 | 0.2837 | 0.3042 | 0.6445 | 0.3474 | 0.4467 | 0.4416 | 0.7461 | 8.8144 | 1.2648 | 0.6795 | 1.0425 |
| HR8 | 0.2394 | 0.1869 | 0.3868 | 0.0918 | 0.1301 | 0.5020 | 0.1877 | 1.4147 | 0.1851 | 0.0000 | 0.3445 |
| HR9 | 0.1446 | 0.5441 | 0.3930 | 0.2773 | 0.3666 | 0.2013 | 0.3715 | 21.4618 | 1.5051 | 0.6076 | 2.3205 |
| HR10 | 0.2687 | 0.3374 | 0.5340 | 0.1620 | 0.2084 | 0.3769 | 0.2588 | 2.2449 | 0.4741 | 0.1405 | 0.4705 |
| HR11 | 0.3272 | 0.3800 | 0.7258 | 0.1642 | 0.2108 | 0.4552 | 0.2493 | 1.9109 | 0.4289 | 0.0000 | 0.4460 |
| CV% | 28.914 | 64.625 | 38.682 | 45.433 | 44.866 | 29.756 | 47.710 | 82.626 | 64.318 | 112.781 | 76.423 |

Table S8 IC_50_ of antioxidant assays of different forms for 11 batches of *C. tubulosa* samples

| DPPH | | | | O_2_^-^• | | | OH• | | |
| --- | --- | --- | --- | --- | --- | --- | --- | --- | --- |
| Batches | H (mg/mL) | WE (mg/mL) | HR (mg/mL) | H (mg/mL) | WE (mg/mL) | HR (mg/mL) | H (mg/mL) | WE (mg/mL) | HR (mg/mL) |
| S1 | 0.07 | 0.05 | 0.17 | 2.03 | 21.09 | 6.86 | 1.32 | 0.84 | 1.43 |
| S2 | 0.80 | 0.28 | 2.70 | 77.64 | 51.99 | 367.40 | 4.11 | 2.11 | 8.95 |
| S3 | 1.87 | 0.73 | 5.38 | 165.50 | 66.73 | 748.80 | 0.79 | 0.77 | 1.97 |
| S4 | 0.33 | 1.23 | 0.43 | 26.94 | 32.52 | 16.39 | 1.81 | 0.60 | 0.92 |
| S5 | 0.33 | 0.42 | 0.24 | 31.31 | 60.87 | 30.82 | 1.10 | 1.47 | 2.11 |
| S6 | 0.21 | 6.16 | 3.44 | 6.31 | 282.70 | 11.08 | 1.15 | 1.52 | 0.91 |
| S7 | 0.04 | 4.11 | 3.82 | 2.93 | 43.53 | 24.59 | 0.88 | 0.32 | 0.48 |
| S8 | 0.08 | 1.20 | 37.80 | 7.96 | 9.01 | 843.90 | 2.97 | 10.21 | 27.65 |
| S9 | 0.27 | 0.16 | 0.43 | 1.54 | 0.98 | 8.55 | 0.92 | 0.45 | 3.96 |
| S10 | 0.57 | 0.73 | 2.87 | 3.70 | 31.79 | 7.35 | 4.04 | 2.46 | 7.09 |
| S11 | 0.74 | 1.81 | 1.11 | 9.80 | 27.67 | 86.08 | 9.29 | 3.43 | 7.77 |

The regression equations obtained using the PLSR model are as follows:

$$Y_{DDPH\left( H \right)}=0.436+0.401\times A_{1}+0.009\times A_{2}+0.118\times A_{3}-0.323\times A_{4}-0.214\times A_{5}-0.162\times A_{6}-0.146\times A_{7}+0.002\times A_{8}-0.032\times A_{9}-0.045\times A_{10}-0.064\times A_{11}$$

$$Y_{DDPH\left( WE \right)}=-1.749+4.728\times A_{1}-1.41\times A_{2}-2.617\times A_{3}+30.001\times A_{4}+17.178\times A_{5}+2.266\times A_{6}+4.638\times A_{7}-0.125\times A_{8}-0.92\times A_{9}-5.004\times A_{10}-0.882\times A_{11}$$

$$Y_{DDPH\left( HR \right)}=33.978-35.798\times A_{1}-6.303\times A_{2}-14.659\times A_{3}-14.508\times A_{4}-9.46\times A_{5}+16.98\times A_{6}-4.839\times A_{7}-0.114\times A_{8}-2.183\times A_{9}-0.596\times A_{10}-0.646\times A_{11}$$

$$Y_{O_{2}^{-}\bullet\left( H \right)}=-58.008+53.591\times A_{1}+1.838\times A_{2}+15.334\times A_{3}-13.466\times A_{4}-3.532\times A_{5}+3.043\times A_{6}+0.803\times A_{7}+0.081\times A_{8}-1.314\times A_{9}-0.999\times A_{10}-4.785\times A_{11}$$

$$Y_{O_{2}^{-}\bullet\left( WE \right)}=-154.825+258.402\times A_{1}-12.282\times A_{2}-7.022\times A_{3}+168.987\times A_{4}+137.501\times A_{5}+276.402\times A_{6}+6.442\times A_{7}-0.927\times A_{8}+13.075\times A_{9}-33.43\times A_{10}-3.128\times A_{11}$$

$$Y_{O_{2}^{-}\bullet\left( HR \right)}=746.969-424.987\times A_{1}+22.591\times A_{2}+79.121\times A_{3}-569.781\times A_{4}-331.707\times A_{5}-50.316\times A_{6}-267.672\times A_{7}-2.33\times A_{8}-57.544\times A_{9}-28.671\times A_{10}-12.60\times A_{11}$$

$$Y_{OH\bullet\left( H \right)}=16.473-3.91\times A_{1}-0.074\times A_{2}-0.642\times A_{3}-2.081\times A_{4}-2.569\times A_{5}-1.797\times A_{6}-1.237\times A_{7}-0.021\times A_{8}-0.334\times A_{9}-0.186\times A_{10}-1.554\times A_{11}$$

$$Y_{OH\bullet\left( WE \right)}=8.595-6.564\times A_{1}-0.106\times A_{2}-0.578\times A_{3}-5.923\times A_{4}-5.011\times A_{5}-2.882\times A_{6}-2.999\times A_{7}-0.019\times A_{8}-0.922\times A_{9}-0.524\times A_{10}-0.145\times A_{11}$$

$$Y_{OH\bullet\left( HR \right)}=27.651-26\times A_{1}-2.138\times A_{2}-9.267\times A_{3}-13.308\times A_{4}-9.332\times A_{5}+12.981\times A_{6}-7.124\times A_{7}-0.052\times A_{8}-2.262\times A_{9}-0.588\times A_{10}-0.276\times A_{11}$$

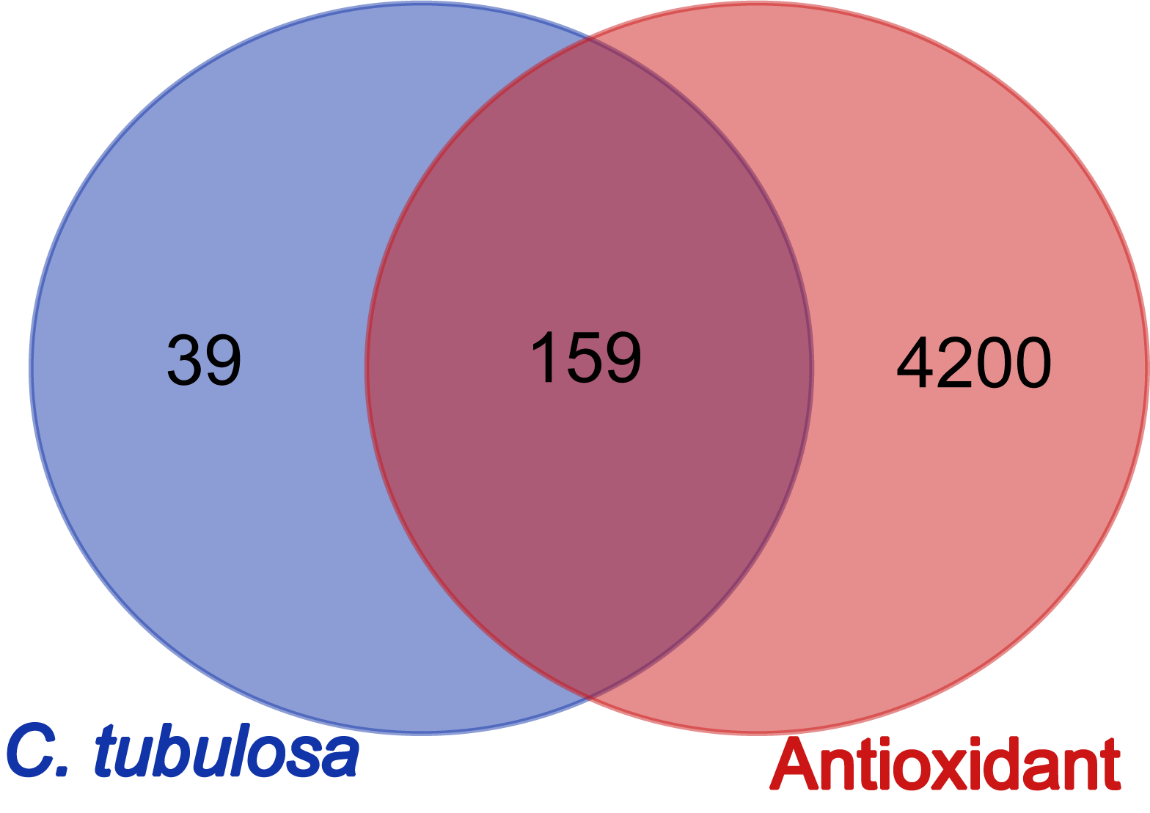


Figure S1: Venn diagram. There were 159 key gene targets between *C. tubulosa*-related targets and antioxidant-related targets.
